# Comparative study of phenolic compounds, vitamin E, and fatty acids compositional profiles in black seed-coated soybeans (*Glycine Max* (L.) Merrill) depending on pickling period in brewed vinegar

**DOI:** 10.1186/s13065-017-0298-9

**Published:** 2017-07-18

**Authors:** Ill-Min Chung, Jin-Young Oh, Seung-Hyun Kim

**Affiliations:** Department of Crop Science, College of Sanghuh Life Science, Konkuk University, 120 Neungdong-ro, Gwangjin-gu, Seoul, 05029 Republic of Korea

**Keywords:** Pickled soybeans, Phenolic compounds, Vitamin E, Fatty acids, Vinegar pickling

## Abstract

**Background:**

Pickled soybeans or vinegar beans have long been used as a folk remedy and also a supplemental nutritional source in Korea. In general the pickling process in vinegar improves the digestibility of soybeans as well as increases the availability of various (non-)nutrients in soybeans. However, detailed information about the changes in functional substances such as (poly)phenolic compounds, vitamin E, and fatty acids (FAs) in soybeans during the pickling process is quite limited. Therefore, this study aims to investigate the changes in the selected phenolic compounds, vitamin E, and FAs in soybeans as a function of the pickling time.

**Results:**

The sum of the total phenolics in both the pickled soybeans and the pickling solutions increased by as much as 47% after pickling. Naringenin, vanillin, and catechin were the major phenolics observed in the pickled soybeans and pickling solutions. The total vitamin E content in the pickled soybeans decreased by 23% after pickling, although no vitamin E molecules were found in the pickling solution. γ–Tocopherol was abundant in the untreated soybeans, but decreased by ~29% after pickling. Both the total and major FA contents varied by less than 1% during the pickling period. In this study, a 10–20 day pickling period may be considered suitable in terms of retention of functional substances in the pickled soybeans, such as selected phenolics, vitamin E, and FAs.

**Conclusions:**

Our findings provide basic information and insight into the production of functional compounds in soybeans upon immersing in brewed vinegar, and also may be helpful toward improving the health-functionality of soybean-based foods in the food industry.

**Graphical abstract:**
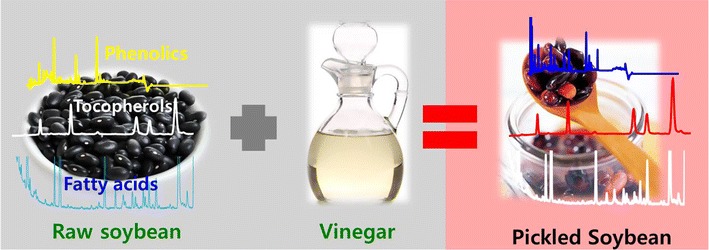
How to change bioactive compounds during the pickling process?

**Electronic supplementary material:**

The online version of this article (doi:10.1186/s13065-017-0298-9) contains supplementary material, which is available to authorized users.

## Background

Soybeans are widely consumed as a dietary source of high quality proteins as well as lipids. A number of nutritionally functional substances found in soybeans, such as isoflavones, saponins, phytic acid, anthocyanins, phytosterols, and dietary fiber, are known to have various health-promoting benefits [1, 2]. For example, soy isoflavones have been reported to have both antioxidant and hormonal activities, which may act to decrease the incidence of certain cancers, cardiovascular disease, and osteoporosis [3, 4]. However, despite the various health benefits of soybeans, the consumption of raw soybeans is limited because of their unpleasant bean flavor, bitter taste, and interference with digestion/absorption caused by anti-nutritional factors such as trypsin inhibitors. Therefore, soybeans have been converted into various soy-based foods after processing or cooking by methods such as heating, fermentation, and germination [1, 5, 6].

Pickled soybeans, or vinegar beans, have long been used as a folk remedy in Korea. They are prepared by immersing soybeans in vinegar for certain periods (pickling). In particular, monks who eat raw foods usually consume pickled soybeans as a supplemental nutritional source [7]. According to prior studies [8–11], the pickled soybean exerts various physiological functions that include relieving fatigue and preventing high blood pressure, and demonstrates hypoglycemic, anticancer, antiproliferative, antiobesity, and antioxidant activities.

Because the pickling process inactivates the anti-nutritional factors (i.e., the trypsin inhibitor) in soybeans, the digestibility of pickled soybeans is usually improved compared to that of raw soybeans. Moreover, because acid hydrolysis during pickling increases the availability of various (non-)nutrients in soybeans, the intake of pickled soybeans significantly improves the digestion/absorption rate in vivo or in vitro. In comparison to raw soybeans, the free amino acids in pickled soybeans increase threefold, and the in vitro digestibility of the soybean protein increases by 22% after pickling [1].

The pickling process simultaneously alters the composition and content of the functional substances in soybeans. Several prior studies [12–14] have described the changes in the isoflavones content and antioxidant activity of soybeans during the pickling process. The total isoflavones, particularly the aglucone types, in soybeans increase significantly as a result of pickling. Likewise, the antioxidant activity, nitrate-scavenging ability, and electron-donation ability also increase upon pickling, relative to raw soybeans [15, 16]. However, to our knowledge, detailed information about the changes in other functional substances such as (poly)phenolic compounds, vitamin E, and fatty acids (FAs) in soybeans during the pickling process is quite limited. Therefore, this study aims to investigate the changes in a selection of 23 phenolic compounds, the vitamin E group including 4 tocopherols and 4 tocotrienols, and 37 FAs in soybeans as a function of the pickling time (0–30 days). These results provide basic information about the changes in functional substance contents in soybeans during the pickling process, and may also be helpful toward improving the health-functionality of soybean-based foods in the food industry.

## Methods

### Soybeans, vinegar, and chemicals

The soybean cultivar, *seoritae*, used in this study was obtained from the Rural Development Administration in Korea. The *seoritae* has a black seed coat and yellow cotyledon color, and the weight of 100 seeds was 11.75 ± 0.13 g. A common brewed, fermented, malt-type vinegar with a total acidity of 6–7% (Ottogi Foods Industries Ltd., Gyeonggi-Do, Korea) was bought from a local market in Seoul, Korea, and was then used for pickled soybean production. All solvents used for extraction and instrumental analysis of phenolic compounds, vitamin E, and FAs were of HPLC or analytical grade. Methanol, ethanol, and isooctane were purchased from Fisher Scientific Korea, Ltd. (Seoul, Korea), and acetonitrile was purchased from Merch KGaA (Darmstadt, German). Acetic acid and hexane were purchased from J. T. Baker (HPLC grade, Phillipsburg, NJ, USA), and benzene, heptane, and potassium hydroxide were purchased from Junsei (Tokyo, Japan). Ascorbic acid was purchased from Sanchun Chemical Co. (Gyeonggi-Do, Korea), and 0.1 N hydrochloric acid, sulfuric acid, and sodium sulfate (anhydrous) were from Daejung Chemical & Materials Co., Ltd. (Gyeonggi-Do, Korea). The 2,2-dimethoxypropane (DMP) and dichloromethane were received from Sigma-Aldrich Corp. (Seoul, Korea). All chemical standards (STDs) used in this study were obtained from Sigma-Aldrich Corp. Phenolic STDs were normally dissolved in methanol. Those that were sparingly soluble in methanol were first dissolved in dimethyl sulfoxide and then diluted with methanol. Each tocopherol and tocotrienol STD was dissolved in isooctane. The 37 fatty acid methyl esters (FAME) standard mixtures and caproic (C6:0), pentadecanoic (C15:0), oleic (C18:1, n9, *cis*), linoleic (C18:2 n6), arachidonic (C20:4 n6), heneicosanoic (C21:0), and docosahexaenoic (C22:6 n3) acids were dissolved in dichloromethane. The C15:0 was used as an internal standard (IS), and other FA STDs were used for the identification of individual FAs in the prepared samples.

### Pickled soybean preparation

The detailed preparation of pickled soybeans was described in our prior study [12]. Soybeans (10 g) were pickled in the brewed vinegar (30 mL) for 1, 5, 10, 20, and 30 days (*n* = 3, each day), and raw soybeans were used as the control. All specimens were stored at room temperature. In each pickling treatment, the pickled soybeans were first separated from the pickling solution, lyophilized (−45 °C, 3 days), and pulverized before analysis. After each pickling treatment, the pickling solution volume was restored to 30 mL using the same vinegar used for pickled soybean production. All samples were stored in a freezer at −70 °C until analysis.

### Extraction and analysis of phenolic compounds

Each pulverized sample (0.5 g) was extracted with acidified acetonitrile (10 mL acetonitrile and 2 mL 0.1 N hydrochloric acid) and shaken at ~0.5×*g* (i.e., 200 rpm) for 2 h at room temperature using a shaker (Green-SSeriker, Vision Scientific Co., Ltd., Gyeonggi-Do, Korea). After shaking, the extracted samples were filtered through Whatman filter paper (No. 42, 110 mm diameter, GE Healthcare Co., Little Chalfont, UK), and the filtrates were evaporated via vacuum rotary evaporator at 35 °C (EYELA SB-1200, Tokyo Rikakikai Co., Ltd., Tokyo, Japan). The concentrated samples were reconstituted with 80% methanol (5 mL) and filtered through a 0.22-μm membrane syringe filter (CHOICE 13 mm, PTFE, Thermo Scientific, Waltham, MA, USA). For the analysis of the phenolic compounds in the pickling solution, each pickling solution was diluted fivefold with 80% methanol and then filtered through the syringe filter after centrifugation to remove soybean matrix particles produced by the pickling process (4 °C, ~2200×*g*, 10 min, VS-6000CFi, Vision Scientific Co., Ltd.) [17]. An Agilent 1290 Infinity Binary UHPLC system coupled with a diode array detector (Agilent Co., Ltd., Seoul, Korea) was used for the phenolic compound analysis. A reverse-phase column (Accucore C18, 100 mm × 2.1 mm, 2.6 μm, Thermo Scientific, USA) was used to separate the phenolics in each sample. The injection volume was 1 μL, and the flow rate was 0.5 mL min^−1^. The mobile phase consisted of 0.1% glacial acetic acid in water (solvent A) and 0.1% glacial acetic acid in acetonitrile (solvent B). The gradient condition of the mobile phase was as follows: 0 min: 98% A, 2% B; 0.5 min: 95% A, 5% B; 2.2 min: 90% A, 10% B; 5 min: 85% A, 15% B; 7.5 min: 84.3% A, 15.7% B; 8 min: 83.4% A, 16.6% B; 9 min: 82.2% A, 17.8% B; 9.5 min: 76.1% A, 23.9% B; 14 min: 55% A, 45% B; 15 min: 0% A, 100% B; 15.5 min: 0% A, 100% B; 16 min: 98% A, 2% B; 22 min: 98% A, 2% B. The total analysis time was 25 min. The UV wavelength was set at 280 nm [18]. The representative chromatograms of phenolic compounds in samples of interest are shown in Additional file 1: Figures S1 and S2.

### Extraction and analysis of vitamin E

Vitamin E molecules, including 4 tocopherols and 4 tocotrienols, were extracted by a previously reported method [17]. For the purpose of vitamin E extraction, the sample (1 g) and ascorbic acid (0.1 g) were gently agitated in ethanol (20 mL) using a water bath/shaker (80 °C, 160 rpm, 10 min). Then, saturated potassium hydroxide solution (300 µL) was added to the extract and agitation was continued in the water bath/shaker (80 °C, 160 rpm, 18 min) to ensure saponification. Afterward, a sample aliquot was cooled on crushed ice for 15 min. Hexane and water (10 mL each) were added to the sample aliquot, centrifuged at 4 °C, ~2000×*g* for 5 min, and then the supernatant (hexane layer) was collected. Additional hexane (10 mL) was added to the residual sample, which was similarly processed and collected after centrifugation. This process was performed a total of 3 times. The collected hexane layer (~30 mL) was washed twice with distilled water (10 mL) and centrifuged at 4 °C, ~2200×*g* for 10 min. Then, the water layer was separated and removed. The remaining hexane layer was filtered through a pad of anhydrous sodium sulfate before concentration on a vacuum rotary evaporator at 35 °C. Finally, the residue was reconstituted in isooctane (1 mL) and stored in an amber vial. The extraction of vitamin E from the pickling solution (1 mL) was performed using the same extraction procedure as for the untreated and pickled soybeans. Vitamin E analysis was accomplished with an Agilent 7890B GC-flame ionization detector (GC-FID) system. A capillary column (CP-SIL 8 CB, 50 m × 0.32 mm, 0.25 μm) was used to separate vitamin E molecules in the samples. The injection volume was 1 μL at a ratio of 1:20 in split mode. The nitrogen carrier gas was set at 25 mL min^−1^, and the flame gas was comprised of hydrogen (25 mL min^−1^) and air (400 mL min^−1^). Both the inlet and detector temperatures were set at 290 °C. The initial oven temperature was 220 °C for 2 min, and was increased to 290 °C at a rate of 5 °C min^−1^, then was kept for 14 min. Finally, the oven temperature was increased to 300 °C at a rate of 10 °C min^−1^ and held for 10 min so that the total analysis time was 41 min [17]. The representative chromatograms of vitamin E in samples of interest are shown in Additional file 1: Figures S3 and S4.

### Quantification of phenolic compounds and vitamin E

Each stock solution (500 μg mL^−1^) of phenolic compounds was diluted to the appropriate concentration depending on the phenolic concentration in the samples. All phenolic calibration curves exhibited good linearity (*r* ^2^ ≥ 0.99) in the range of 0.1–50 μg mL^−1^. Vitamin E STDs were dissolved in isooctane at a concentration of 1000 μg mL^−1^ as stock solutions. All vitamin E calibration curves exhibited good linearity (*r* ^2^ ≥ 0.99) in the range of 1 − 200 μg mL^−1^ (Additional file 1: Tables S1, S2). The phenolic compounds and vitamin E in the prepared samples were measured by comparing the retention times of the peaks in the samples against the authentic STD chromatograms. In addition, each phenolic compound and vitamin E STD was added to the sample aliquot to confirm the peak assignments. The limit of detection (LOD) and limit of quantification (LOQ) were calculated by calibration curves prepared according to LOD = 3 × *SD*/*S* and LOQ = 10 × *SD/S*, where *SD* is the standard deviation of the *y*-intercept of the calibration curve, and *S* is the slope of each calibration curve (Additional file 1: Tables S1, S2) [17].

### Derivatization, extraction, and analysis of fatty acids

Prior to the GC-FID measurements, the FAs in the prepared samples were derivatized as FAMEs and simultaneously extracted [19]. The pulverized pickled soybeans (50 mg) were placed in a 2 mL amber vial, and pentadecanoic acid (0.2 mg) was added as an IS. Next, the solvent mixture for FA derivatization and extraction, consisting of heptane (400 μL), and the methylation mixture (680 μL, methanol:benzene:DMP:sulfuric acid = 39:20:5:2, by vol) was added to the amber vial, which was then placed in a water bath/shaker (BF-45SB, Biofree Co., Ltd., Seoul, Korea) for 2 h at 80 °C. Thereafter, the mixture was cooled to room temperature, and the supernatant was transferred into a microcentrifuge tube and centrifuged at ~45×*g* for 2 min. The FA profiles of the untreated and pickled soybeans in the final supernatants were analyzed by GC-FID. The pickling solution (50 µL) was extracted in the same way as described above for the untreated and pickled soybeans.

The FAME analysis was performed with an Agilent 7890B GC-FID system. An Agilent J&W capillary column (HP-INNOWax 19091 N, 30 m × 0.25 mm, 0.25 μm) was used to separate the 37 FAs in the FAME STDs mixture and the samples. The injection volume was 1 μL in the split mode (1:50). Helium carrier gas was set at 10 mL min^−1^, and the flame gas consisted of both hydrogen (35 mL min^−1^) and air (300 mL min^−1^). The initial oven temperature was set at 100 °C for 2 min and increased to 150 °C at a rate of 5 °C min^−1^, then kept for 2 min. Subsequently, the oven temperature was increased to 240 °C at a rate of 5 °C min^−1^ and then kept for 5 min. The inlet and FID temperatures were 230 and 250 °C, respectively, and the total analysis time was 64 min [19]. The representative chromatograms of FAs in the samples of interest are shown in Additional file 1: Figure S5.

### Quantification of fatty acids

The mixture of 37 FAME STDs (1 mL) was dissolved in dichloromethane (9 mL). Each FA in a sample aliquot was identified by comparing its retention time against the FAME STDs mixture. Certain FA standards, such as C6:0, C18:1n-9, C18:2n-6, C20:4n-6, C21:0, and/or C22:6n-3, were added to sample aliquots to check the accuracy of the peak assignments. The FA content (mg g^−1^, dry basis) in each sample was calculated using the method in the Korean Food Standards Codex issued by the Ministry of Food and Drug Safety while considering the conversion and response factors of the individual FA [20].

### Statistical analysis

In this study, the pickled soybeans were prepared in triplicate, and sample extractions and instrumental analyses were conducted in duplicate. The statistical analysis was performed with SAS software (version 9.3, SAS Institute, Inc.) using the general linear model procedure. In addition, the least significant difference (LSD) test was conducted at the 0.05 probability level to determine the differences between the means among the samples.

## Results and discussion

### Changes in the phenolics content in soybeans during pickling

Table 1 shows the changes in the phenolics content in the untreated soybeans, pickled soybeans, and pickling solutions, depending on the pickling period. The total phenolic compounds content in the untreated soybeans was 261.7 μg g^−1^. Among the 23 phenolic compounds tested in this study, only eight (protocatechuic acid, *m*-coumaric acid, *t*-cinnamic acid, catechin, naringin, quercetin, naringenin, and vanillin) were found in the untreated soybeans. Vanillin was the most abundant phenolic compound (144.3 μg g^−1^) found in the untreated soybeans, accounting for 55% of the total, and catechin (53.0 μg g^−1^) and naringin (38.6 μg g^−1^) were the next most abundant. With respect to the phenolic compound type, the phenolic acids and flavonoid groups represented 61 and 39%, respectively, of the total phenolics content in the untreated soybeans.

**Table 1:** Change in phenolic compounds content in soybeans during the 30-day pickling process (μg g^−1^, dry basis, *P* < 0.05, *n* = 6)

	Untreated soybean (control)	Pickled soybean					LSD _(0.05)_
		Day 1	Day 5	Day 10	Day 20	Day 30	
Phenolic acid							
PR	12.1 ± 1.5^d^	11.7 ± 1.2^d^	14.6 ± 1.9^c^	15.3 ± 2.3^c^	19.4 ± 1.3^a^	17.6 ± 2.5^b^	1.6
*m*C	1.9 ± 0.2^a^	1.5 ± 0.2^c^	1.7 ± 0.2^b^	1.5 ± 0.1^c^	1.4 ± 0.1^cd^	1.3 ± 0.2^d^	0.1
* t*C	2.1 ± 0.3^f^	3.5 ± 0.5^e^	4.7 ± 0.6^d^	5.5 ± 0.5^c^	6.9 ± 0.9^b^	7.9 ± 0.9^a^	0.6
VA	144.3 ± 6.4^a^	94.6 ± 9.7^b^	90.0 ± 6.0^bc^	86.4 ± 3.3^c^	73.2 ± 10.8^d^	72.3 ± 7.5^d^	6.5
Flavonoid							
CA	53.0 ± 1.7^a^	39.6 ± 3.5^b^	37.3 ± 18.7^b^	36.2 ± 5.1^b^	48.5 ± 14.0^a^	49.3 ± 3.7^a^	8.5
NA	38.6 ± 2.4^a^	35.3 ± 2.2^ab^	32.5 ± 4.3^bc^	31.3 ± 2.5 ^cd^	27.9 ± 4.2^d^	26.3 ± 5.2^d^	4.0
QU	7.2 ± 1.6^b^	7.6 ± 1.4^ab^	7.4 ± 1.7^b^	7.3 ± 2.1^b^	8.9 ± 1.0^a^	8.0 ± 2.3^ab^	1.5
NG	2.5 ± 0.5^f^	10.3 ± 0.9^e^	18.1 ± 1.3^d^	25.0 ± 2.3^c^	41.4 ± 2.6^b^	60.3 ± 5.8^a^	2.4
Total phenolics							
	261.7 ± 8.1^a^	204.1 ± 10.0^d^	206.2 ± 24.2^d^	208.4 ± 4.4^d^	227.5 ± 19.2^c^	242.8 ± 17.5^b^	13.3

	Vinegar (control)	Pickling solution					
		Day 1	Day 5	Day 10	Day 20	Day 30	LSD _(0.05)_
Phenolic acid							
PR	ND	13.1 ± 1.6^bc^	15.3 ± 1.2^ab^	14.3 ± 2.4^ab^	17.3 ± 4.5^a^	11.8 ± 2.3^c^	1.7
*m*C	ND	0.9 ± 0.0^c^	1.1 ± 0.2^b^	1.2 ± 0.3^ab^	1.3 ± 0.2^a^	1.3 ± 0.3^a^	0.1
*t*C	ND	1.8 ± 0.1^c^	1.9 ± 0.2^bc^	2.0 ± 0.2^b^	2.3 ± 0.3^a^	2.3 ± 0.3^a^	0.1
VA	ND	14.8 ± 3.6^b^	18.7 ± 4.2^a^	16.7 ± 2.1^ab^	17.6 ± 3.0^a^	14.5 ± 2.7^b^	2.0
Flavonoid							
CA	ND	62.4 ± 9.4 ^cd^	66.3 ± 3.7^c^	60.1 ± 8.5^d^	82.5 ± 8.4^b^	93.4 ± 4.8^a^	4.7
NA	ND	4.1 ± 0.4^b^	4.8 ± 1.1^a^	4.7 ± 0.6^a^	5.1 ± 0.5^a^	5.0 ± 0.4^a^	0.4
QU	ND	TR	TR	4.3 ± 0.2	5.6 ± 0.7	5.9 ± 0.2	–
NG	ND	1.8 ± 0.9^c^	1.8 ± 0.1^c^	2.0 ± 0.2^c^	3.0 ± 0.1^b^	3.6 ± 0.2^a^	0.3
Total phenolics							
	ND	102.2 ± 11.3^c^	110.6 ± 6.4^b^	108.9 ± 12.6^bc^	135.6 ± 5.0^a^	140.9 ± 9.2^a^	6.0

*ND* not detected, *TR* trace level concentrations, *PA* phenolic acid, *F* flavonoid, *PR* protocatechuic acid, *m*C *m*-coumaric acid, *t*C *t*-cinnamic acid, *CA* catechin, *NA* naringin, *QU* quercetin, *NG* naringenin, *VA* vanillin

^a–f^ Values with different superscripts differ significantly according to pickling period (*P*< 0.05)

**Fig. 1:**
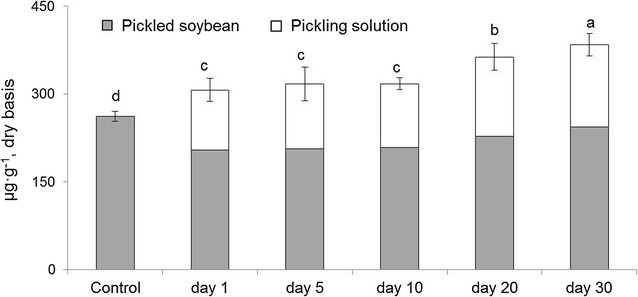
Sum of total phenolics content in the pickled soybeans and pickling solution as a function of pickling period (*P* < 0.05, *n* = 3). Control: untreated soybeans; day 1–30: the pickling period

**Fig. 2:**
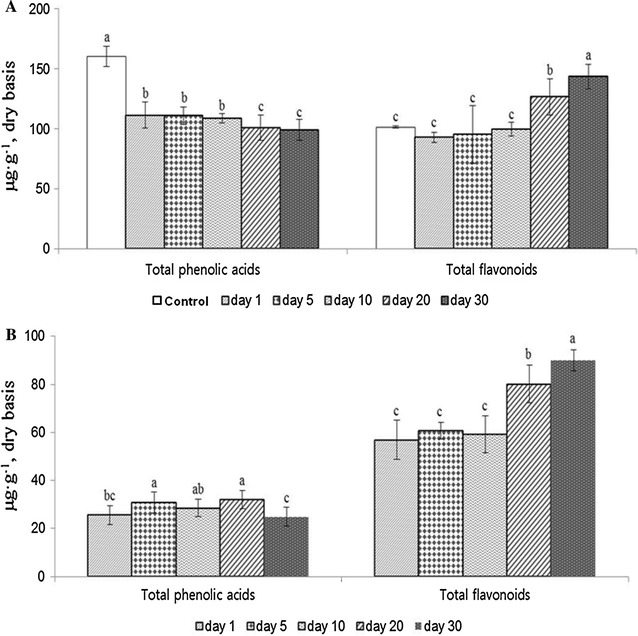
Changes in the total phenolic acids and total flavonoids in the pickled soybeans (**A**) and the pickling solution (**B**) according to pickling period over 30 days (*P* < 0.05, *n* = 3). Control: untreated soybeans; day 1–30: the pickling period

During the pickling process, the sum of the total phenolics in the pickled soybeans and the pickling solution increased as the pickling period was prolonged. The sum of the total phenolics increased by 47% (from 261.7 to 383.8 μg g^−1^) after 30 days of pickling (Fig. 1). For the pickled soybeans, the phenolic composition was the same as in the untreated soybeans; however, the phenolic content changed significantly with respect to the pickling period (Table 1). The total phenolics content in the pickled soybeans decreased by 7–22% after 30 days of pickling, in comparison to that of the untreated soybeans. Furthermore, the total phenolic acids were decreased by 35%, whereas the total flavonoids were increased by 42% compared to those in the untreated soybeans over the 30-day period (Fig. 2). Naringenin, vanillin, and catechin were the most abundant phenolics found in pickled soybeans. In particular, the naringenin content in the pickled soybeans significantly increased from 2.5 to 60.3 μg g^−1^, whereas the vanillin content decreased by about 50% from 144.3 to 72.3 μg g^−1^ after 30 days (Table 1, *P* < 0.05).

The brewed vinegar used for pickling the soybeans did not initially contain phenolic compounds; however, certain phenolics were leached (or extracted) from the soybeans into the pickling solution during their immersion. The total phenolic compounds found in the pickling solutions were 39–54% of the total phenolics present in the untreated soybeans, and the 30th day pickling solution had the largest amount of total phenolics (140.9 μg g^−1^) in this study. The total flavonoids in the pickling solution accounted for 26–41% of the total phenolics in the untreated soybeans, and these amounts continually increased with respect to the pickling period over the 30 days. In contrast, the total phenolic acids in the picking solutions were not influenced by the pickling period; these accounted for ≤15% of the total phenolics in untreated soybeans (Table 1; Fig. 2). In addition, catechin was the predominant phenolic type found in the pickling solution for the entire pickling period, and its content was 55–66% of the total phenolics found in the pickling solutions (Table 1).

Certain food processing or cooking conditions, including soaking, heating, pressure, fermenting, acidifying, or deforming, affect the content and composition of nutrients and nutraceuticals in soybeans and soy foods [12, 17]. For example, the total isoflavones content, as well-known nutraceuticals in soybeans, was unchanged during fermentation; however, their composition was significantly altered (e.g., the aglucone types increased) [21]. In another study, the contents of the phenolic compounds (–8%), vitamin E (–3%), and amino acids (+10%) in soybeans were changed during soybean-rice cooking in an electric pressurized rice cooker (heating + pressure) [17]. In addition, vitamin E and isoflavones in soybeans increased to 32.4 and 27.9%, respectively, after germination for 24 h [22].

Pickled soybeans are prepared by immersion in vinegar for several days (usually ~10 days) to remove their undesirable bean flavor and the bitter taste of raw soybeans, and to increase the in vivo digestion/absorption of soy proteins with high molecular weights [1]. Hence, the acidic condition of the pickling process affects the content and composition of various nutrients and nutraceuticals in the pickled soybeans and the pickling solution. In a prior study [15], the total phenols content in pickled soybeans was preserved at 63–66% of the total phenols present in the raw soybeans. In addition, the total phenols content in soybeans pickled for more than 4 days was increased due to the acid hydrolysis of complicated polyphenols in the pickled soybeans. Compared to the level in untreated soybeans, the total flavonoids content decreased by ~50% after pickling for 24 h; thereafter, its variation was insignificant over two additional weeks of pickling [16]. The decrease in the total flavonoids content in pickled soybeans may be influenced by the decrease in the content of anthocyanins, which are abundant in black-coated soybeans [15]. However, unlike the total flavonoids content in pickled soybeans, the isoflavones (in particular, the aglucone types)—another representative class of soy flavonoids—in pickled soybeans increase over time while pickling in vinegar [1, 7, 12, 15]. The total isoflavones in both the pickled soybeans and the pickling solution after 30 days of pickling peaked at 93%, an increase compared to those in the untreated soybeans. Furthermore, although aglucone-type isoflavones are not found in untreated soybeans, aglucones were found at a maximum level of 154.6 μg g^−1^ in the pickled soybeans after 30 days immersion. However, malonyl and acetyl glucoside-types decreased by 17–41% after the pickling process [7].

Hitherto, to our knowledge, there has been no comprehensive report concerning the changes in the levels of individual phenolic compounds during the pickling process. In this study, the sum of the total phenolic contents in the pickled soybeans and the pickling solution also increased by as much as 47% after pickling for 30 days (Fig. 1), which is consistent with a prior study that reported an increase in the total phenol content in pickled soybeans as a function of the pickling period. In this study, the increase in the total phenolics content was associated with an increase in the total flavonoids content (in particular, naringenin and catechin) in both the pickled soybeans (*r* ^2^ = 0.931, *P* < 0.0001) and the pickling solutions (*r* ^2^ = 0.561, *P* < 0.0001). In contrast, the total phenolic acids content in the pickled soybeans decreased as the pickling period was extended, and the vanillin content was highly associated with the decrease in the total phenolic acids in the pickled soybeans (*r* ^2^ = 0.986, *P* < 0.0001).

The variation of phenolics content during the pickling process may be associated with the change in soybean texture under the acidic conditions and/or incomplete germination (or sprouting). In prior studies [1, 7], the acidity of the pickling solution was kept low (pH ≤ 4.0) during the pickling period. In fact, in comparison to untreated soybeans, the pickled soybeans were ten times softer, and their strength was reduced after pickling [1, 7]. Besides, the smooth texture of the soybeans after germination increased the soy protein content by more than 40% in comparison to the raw soybeans, owing to the improved extraction efficiency of the soy proteins [23]. Phenolic compound contents are known to be enhanced in sprouted or germinated grains such as wheat and soybeans, by either de novo biosynthesis or enzymatic hydrolysis [24, 25]. For example, the total phenols, total flavonoids, and ferulic acid contents in wheat germinated for 5 days were increased by 9.9, 30.7, and 21.6%, respectively. Also, the content of vitamin E was increased more than twofold in germinated wheat [24].

In the present study, the mean acidity of all pickling solutions was approximately pH 4.0. In addition, the soybeans were incompletely germinated (signified by the appearance of hypocotyls of ~0.5 mm) during the 30-day pickling process. These factors may have caused the soybean texture to soften. In this way, the improved extraction efficiency and/or *de novo* biosynthesis caused by the incomplete sprouting may have altered the total abundance of phenolic compounds in the pickled soybeans and pickling solutions. Also, acid hydrolysis during the pickling process may have affected the content of phenolic compounds in the pickled soybeans [14].

### Changes in vitamin E content in soybeans during pickling

Vitamin E molecules are known as important sources of lipid-soluble antioxidants that prevent lipid oxidation, and as essential nutrients for human health [26]. α-, β-, γ-, and δ-Tocopherols are typically found in soybeans, with γ-tocopherol as the major component. The α-tocopherol is known to have the highest biological activity, whereas the γ-tocopherol shows the highest antioxidant activity [27]. In general, the vitamin E content in foods is affected by food processing and cooking conditions such as fermentation, heating, flaking, degumming, bleaching, and deodorizing [27]. For example, microwave heating decreases the vitamin E content in soybeans by approximately 40% [28]. Also, 7% of the total vitamin E in soybeans were decomposed during the cooking of soybean-rice mixtures in an electric rice cooker [17].

In the case of detailing vitamin E variations during pickling, there are no comprehensive reports to date. Only one prior study reported that the vitamin B_1_ and C contents in soybeans declined during pickling [16]. In this study, the vitamin E compositions and the major vitamin E in (pickled) soybeans are similar to the prior report [22].

Table 2 shows the variations in the vitamin E content depending on the soybean pickling period. α-, β-, γ-, and δ-Tocopherols were found in the untreated soybeans, with a total vitamin E content of 156.0 μg g^−1^, which can be expressed as 42.2 μg g^−1^ of α-tocopherol equivalents. In the untreated soybeans, γ-tocopherol is the most abundant type, accounting for 69% of the total vitamin E. After pickling for 1 day, the total vitamin E increases in the soybeans by ~22%; however, after pickling for 30 days, the total vitamin E in the pickled soybeans decreases by 23% (Table 2). γ-Tocopherol is also the most abundant type in pickled soybeans, accounting for 59–67% of the total vitamin E, although the γ-tocopherol content decreases by ~29% during the pickling period. No vitamin E molecules were present in either the brewed vinegar or any of the pickling solutions in this study. γ-Tocopherol was only detected below the LOD in certain pickling solutions (see Additional file 1: Figure S4).

**Table 2:** Change in vitamin E content in soybeans during the 30-day pickling process (μg g^−1^, dry basis, *P* < 0.05, *n* = 6)

Pickled soybean							
	Untreated soybean (control)	Day 1	Day 5	Day 10	Day 20	Day 30	LSD _(0.05)_
α-tocopherol	8.8 ± 1.1^bc^	10.6 ± 0.8^a^	8.4 ± 1.0 ^cd^	8.0 ± 0.7 ^cd^	10.4 ± 2.0^ab^	7.2 ± 1.5^d^	1.6
β-tocopherol	1.3 ± 0.1^b^	1.5 ± 0.1^a^	1.3 ± 0.0^b^	1.2 ± 0.1^b^	1.3 ± 0.1^b^	0.9 ± 0.1^c^	0.2
γ-tocopherol	108.1 ± 7.1^a^	118.4 ± 7.7^a^	110.2 ± 4.1^a^	106.6 ± 1.7^a^	95.2 ± 9.7^b^	77.2 ± 12.3^c^	11.4
δ-tocopherol	37.7 ± 3.3 ^cd^	60.2 ± 1.5^a^	45.9 ± 2.5^bc^	41.0 ± 1.5 ^cd^	53.2 ± 6.0^ab^	34.4 ± 2.6^d^	8.2
Total	156.0 ± 10.9^b^	190.8 ± 8.3^a^	165.8 ± 6.4^b^	156.8 ± 2.7^b^	160.1 ± 13.8^b^	119.7 ± 15.1^c^	18.1
α-tocopherol equivalent	42.2 ± 3.3^b^	47.3 ± 3.1^a^	42.5 ± 2.3^b^	40.9 ± 1.2^b^	40.0 ± 5.0^b^	31.1 ± 5.3^c^	4.1

α-tocopherol equivalent is based on the biological activity of vitamin E vitamers and is calculated as follows: α-tocopherol equivalent = sum of α-tocopherol + (β-tocopherol × 0.4) + (γ-tocopherol × 0.3) + (δ-tocopherol × 0.01)

^a–d^ Values with different superscripts differ significantly according to pickling period (*P*< 0.05)

The vitamin E content is also influenced by germination. The total vitamin E content increases by more than double in soy germs and wheat sprouts after germination. Among the vitamin E molecules, the α–tocopherol, in particular, significantly increases after germination [22, 24, 29]. In this study, the change in soybean texture and/or incomplete germination during the pickling period may have affected the increase in the total vitamin E in the pickled soybeans after pickling for 1 day. In addition, the decrease in the total vitamin E in the pickled soybeans may be associated with the decomposition of the γ-tocopherol (*r* = 0.91, *P* < 0.0001) caused by (acetic) acid hydrolysis during pickling. According to a prior study [27], the α-tocopherol is more stable toward degradation by bleaching, whereas the α-tocopherol in oils is less stable to microwave heating. In this study, the γ-tocopherol was the most sensitive to the pickling process by vinegar, whereas the α-tocopherol was relatively stable. Hence, on the basis of our findings, a pickling period of more than 10 days may reduce the antioxidant activity of the vitamin E in the pickled soybeans.

### Changes in the fatty acids content in soybeans during pickling

Table 3 shows the variation in the FAs profile in the untreated and pickled soybeans depending on the pickling period. In both sample groups, linoleic acid was the major FA, accounting for approximately 53% of the total FAs. Oleic, palmitic, α-linolenic, and stearic acids were the next most abundant, comprising ~22, 11, 8, and 5% of the total FA content, respectively. During the soybean pickling period, both the total and major FA contents varied by less than 1%, which was not statistically significant with respect to the pickling period. In addition, in the untreated soybeans and all pickled soybeans, the total unsaturated FA content was ~fivefold larger than the total saturated FA content, and the polyunsaturated fatty acid (PUFA) content was about threefold higher than the monounsaturated fatty acid (MUFA) content (*P* < 0.05). In particular, the n-6 FA content was about sevenfold higher than the n-3 FA content. Also, long chain FAs consisting of 13–21 carbons were the most predominant FA types present in the untreated and pickled soybeans. Finally, no FAs were detected in any of the pickling solutions in this study (Table 3).

**Table 3:** Change in fatty acid content in soybeans during the 30-day pickling process (mg g^−1^, dry basis, *P* < 0.05, *n* = 6)

Pickling period							
	Untreated soybean (control)	Day 1	Day 5	Day 10	Day 20	Day 30	LSD _(0.05)_
C6:0	0.02 ± 0.01^a^	0.02 ± 0.00^a^	0.02 ± 0.01^a^	0.02 ± 0.01^a^	0.02 ± 0.01^a^	0.02 ± 0.00^a^	0.01
C8:0	0.02 ± 0.00^a^	0.01 ± 0.00^b^	0.01 ± 0.03^c^	TR	TR	TR	0.00
C10:0	0.05 ± 0.00^a^	0.04 ± 0.01^ab^	0.04 ± 0.01^bc^	0.04 ± 0.01^bc^	0.03 ± 0.01^c^	0.03 ± 0.01^c^	0.01
C14:0	0.10 ± 0.01^c^	0.10 ± 0.02^c^	0.10 ± 0.01^abc^	0.10 ± 0.01^bc^	0.11 ± 0.01^a^	0.11 ± 0.02^ab^	0.01
C14:1	0.06 ± 0.03^b^	0.09 ± 0.04^ab^	0.10 ± 0.04^a^	0.07 ± 0.05^ab^	0.07 ± 0.04^ab^	0.09 ± 0.05^ab^	0.03
C16:0	16.75 ± 0.85^a^	16.42 ± 1.00^a^	16.48 ± 0.80^a^	16.12 ± 2.97^a^	16.29 ± 0.59^a^	17.35 ± 2.21^a^	1.35
C16:1	0.13 ± 0.01^a^	0.12 ± 0.01^a^	0.12 ± 0.01^a^	0.13 ± 0.01^a^	0.12 ± 0.01^a^	0.13 ± 0.02^a^	0.01
C18:0	6.81 ± 0.40^a^	6.65 ± 0.54^a^	6.74 ± 0.37^a^	6.86 ± 0.30^a^	6.72 ± 0.27^a^	7.09 ± 1.04^a^	0.45
C18:1n9 cis + trans	32.05 ± 1.97^ab^	30.97 ± 1.39^ab^	31.37 ± 1.60^ab^	31.82 ± 1.01^ab^	30.71 ± 1.379^b^	32.66 ± 4.03^a^	1.75
C18:2 n-6 cis	78.27 ± 4.45^ab^	76.07 ± 3.90^ab^	75.73 ± 3.07^ab^	78.44 ± 2.16^ab^	77.16 ± 2.27^b^	78.11 ± 4.93^a^	2.95
C18:3 n-3	11.53 ± 0.65^a^	11.07 ± 0.57^a^	11.11 ± 0.55^a^	11.43 ± 0.36^a^	10.82 ± 0.81^a^	11.49 ± 1.69^a^	0.72
C20:0	0.49 ± 0.03^a^	0.49 ± 0.04^a^	0.49 ± 0.02^a^	0.51 ± 0.02^a^	0.49 ± 0.01^a^	0.50 ± 0.09^a^	0.04
C20:1 n-9	0.32 ± 0.05^a^	0.33 ± 0.05^a^	0.33 ± 0.06^a^	0.34 ± 0.05^a^	0.34 ± 0.04^a^	0.35 ± 0.05^a^	0.04
C20:2	0.05 ± 0.005^a^	0.04 ± 0.01^b^	0.05 ± 0.01^ab^	0.05 ± 0.00^a^	0.05 ± 0.01^ab^	0.05 ± 0.01^a^	0.01
C20:3 n-6	0.07 ± 0.00^a^	0.06 ± 0.01^a^	0.06 ± 0.01^a^	0.07 ± 0.00^a^	0.06 ± 0.00^a^	0.07 ± 0.02^a^	0.01
C22:0	0.58 ± 0.05^a^	0.59 ± 0.06^a^	0.57 ± 0.03^a^	0.61 ± 0.03^a^	0.58 ± 0.03^a^	0.59 ± 0.15^a^	0.06
C22:2	0.04 ± 0.01^b^	0.04 ± 0.01^ab^	0.04 ± 0.01^b^	0.05 ± 0.02^ab^	0.04 ± 0.01^b^	0.06 ± 0.02^a^	0.01
C24:0	0.21 ± 0.03^b^	0.22 ± 0.03^ab^	0.22 ± 0.02^ab^	0.23 ± 0.02^ab^	0.21 ± 0.02^b^	0.24 ± 0.04^a^	0.02
Total FA	147.54 ± 8.32^a^	143.36 ± 7.30^a^	143.58 ± 5.70^a^	146.88 ± 5.52^a^	143.82 ± 3.72^a^	148.94 ± 14.00^a^	6.62
Calculated value							
∑ SFA	25.03 ± 1.30^a^	24.55 ± 1.60^a^	24.67 ± 1.23^a^	24.49 ± 3.075^a^	24.45 ± 0.87^a^	25.94 ± 3.55^a^	1.79
∑ UFA	122.55 ± 7.06^a^	118.86 ± 5.57^a^	118.96 ± 4.88^a^	122.43 ± 3.020^a^	119.41 ± 2.96^a^	123.03 ± 10.53^a^	5.12
∑ MUFA	32.60 ± 2.01^ab^	31.56 ± 1.38^ab^	31.96 ± 1.61^ab^	32.40 ± 1.061^ab^	31.28 ± 1.38^b^	33.26 ± 4.13^a^	1.79
∑PUFA	89.95 ± 5.10^a^	87.30 ± 4.27^a^	86.99 ± 3.59^a^	90.03 ± 2.457^a^	88.13 ± 2.12^a^	89.78 ± 6.60^a^	3.54
MUFA/PUFA	0.36 ± 0.01^ab^	0.36 ± 0.01^ab^	0.37 ± 0.01^a^	0.36 ± 0.012^ab^	0.35 ± 0.01^b^	0.37 ± 0.02^a^	0.01
∑ n-3 PUFA	11.53 ± 0.65^a^	10.75 ± 0.14^a^	11.08 ± 0.52^a^	11.43 ± 0.36^a^	10.83 ± 0.04^a^	10.83 ± 0.04^a^	0.09
∑ n-6 PUFA	78.33 ± 4.46^a^	76.14 ± 3.90^a^	75.79 ± 3.07^a^	78.50 ± 2.16^a^	77.22 ± 2.27^a^	78.18 ± 4.95^a^	2.95
n-3/n-6	0.15 ± 0.00^a^	0.14 ± 0.01^ab^	0.15 ± 0.00^a^	0.15 ± 0.00^ab^	0.14 ± 0.00^b^	0.14 ± 0.01^a^	0.01
∑ medium (6–12)	0.08 ± 0.01^a^	0.06 ± 0.01^b^	0.06 ± 0.01^bc^	0.06 ± 0.008^cd^	0.05 ± 0.00^d^	0.05 ± 0.01^cd^	0.01
∑ long (13–21)	146.67 ± 8.29^a^	142.49 ± 6.89^a^	142.73 ± 5.68^a^	145.98 ± 5.52^a^	142.99 ± 3.70^a^	148.03 ± 13.83^a^	6.56
∑ very long (≥22)	0.83 ± 0.08^a^	0.85 ± 0.10^a^	0.83 ± 0.05^a^	0.88 ± 0.06^a^	0.83 ± 0.04^a^	0.89 ± 0.21^a^	0.09

∑ *SFA* sum of saturated fatty acids, ∑ *UFA* sum of unsaturated fatty acids, ∑ *MUFA* sum of monounsaturated fatty acids, ∑ *PUFA* sum of polyunsaturated fatty acids, *MUFA/PUFA* sum of monounsaturated fatty acids/sum of polyunsaturated fatty acids, ∑ *n-3 PUFA* sum of omega-3 fatty acids, ∑ *n-6 PUFA* sum of omega-6 fatty acids, *n-3/n-6* sum or omega-3 fatty acids/sum of omega-6 fatty acids, *∑ medium* sum of medium chain fatty acids including 6–12 carbons, ∑ *long* sum of long chain fatty acids including 13–21 carbons, ∑ *very long* sum of long chain fatty acids including more than 22 carbons, *TR* trace level

^a–d^ Values with different superscripts differ significantly according to pickling period (*P*< 0.05)

Soybeans are crucial sources of dietary calories and oil. Their lipid content are ~20%, which are mostly present in the form of triglycerides (~90%) [30]. Palmitic, oleic, and linoleic acids are the major FAs found in soybeans, and linoleic acid, in particular, is the most abundant FA, accounting for ~55% of the total [31]. In general, food processing methods such as heating are known to decrease the nutritional value of foods, although digestibility and the bioavailability of nutrients and nutraceuticals are increased [32]. For example, depending on the soybean heating conditions, the content of linolenic acid was found to decrease by 14–38%, and that of linoleic acid decreased by as much as 8.5% [33]. Germination also affects the FA profiles in soybeans. In a prior study [22], after germinating for 24 h, the saturated FA contents in soybeans were slightly decreased, whereas unsaturated FAs (particularly linolenic acid) were increased from 8 to 21%. Another study [16] showed that pickling did not affect the FA composition and content in soybeans. Linoleic and oleic acids were found as the major FAs in both untreated and pickled soybeans, and their contents varied by ~1% over a 10-day period. In this study, the major FA types were also linoleic and oleic acids, accounting for 53 and 22% of the total FAs, respectively, in both the untreated and pickled soybeans. Furthermore, the major FA contents measured in this study also varied by less than 1% and were statistically independent of the pickling period.

In general, the intake of essential FAs (n-3 and n-6 types) has many health-promoting benefits, having been related to delays in the onset or progress of cardiovascular disease, hypertension, type 2 diabetes, and certain cancers, and to immune system enhancements [34, 35]. For example, n-6 FAs like linoleic acid can serve as precursors to powerful hormone-like compounds that enhance the transport of bioactive compounds [36]. The increase of n-3 FA intake from plant or fish sources is known to decrease the risk of cardiovascular mortality. However, although FAs are essential nutrients, a larger intake of n-6 FAs relative to n-3 FAs is less desirable for human health, because high n-6 FA consumption promotes certain cancers, as well as cardiovascular, inflammatory, and autoimmune diseases [37]. In this study, the n-3/n-6 FA ratio in both the untreated and pickled soybeans was ~1:7 over the entire pickling period, and this ratio was at least twofold lower than the recommended dietary level of 1:1–1:4 [37]. Hence, for the purpose of a desirable nutritional balance, the intake of n-3 FA-rich foods with the pickled soybeans may be required for better human health.

## Conclusions

The vinegar pickling process changes the content and composition of functional substances in soybeans. After the 30-day pickling process, the sum of the total phenolics in the pickled soybeans and the pickling solutions increased by 47%, whereas the total vitamin E content in the pickled soybeans decreased by 23%. The pickling period did not affect the total FA content in the pickled soybeans. Besides, in this study, the saturated FAs (*r* = −0.27, *P* < 0.05) and the n3/n6 ratio (*r* = −0.37, *P* < 0.01) were negatively correlated with the total vitamin E content. The total vitamin E content in the pickled soybeans was associated with the total phenolics in the pickled soybeans (*r* = −0.34, *P* < 0.01) and pickling solution (*r* = −0.49, *P* < 0.0001). A longer pickling period tended to decrease the total vitamin E content (*r* = −0.55, *P* < 0.0001) in the pickled soybeans, whereas it increased the sum of total phenolics (*r* = 0.85, *P* < 0.0001) in both the pickled soybeans and the pickling solution. Therefore, a 10–20 day pickling period may be considered suitable in terms of functional substances in the pickled soybeans, such as selected phenolics, vitamin E molecules, and FAs. The present study may provide insight into the production of the functional compounds measured in pickled soybeans.

## Additional file

**Additional file 1.** Additional tables and figures.

## Abbreviations

DMP: 2,2-dimethoxy propane
FA: fatty acid
FAME: fatty acid methyl ester
FID: flame ionization detector
IS: internal standard
LOD: limit of detection
LOQ: limit of quantification
LSD: least significant difference
MUFA: monounsaturated fatty acid
PUFA: polyunsaturated fatty acid
STD: standard
