# Supplementary material for: Comparative study of phenolic compounds, vitamin E, and fatty acids compositional profiles in black seed-coated soybeans (Glycine Max (L.) Merrill) depending on pickling period in brewed vinegar
Source: Chem Cent J. 2017 Jul 18;11:64. doi: 10.1186/s13065-017-0298-9 (PMC5515724; doi:10.1186/s13065-017-0298-9)
Supplement: Supplementary file 1 — Additional file 1. Additional tables and figures. [file 13065_2017_298_MOESM1_ESM.docx]

**[Supplementary materials]** including 2 Tables and 5 Figures

**Comparative Study of Phenolic Compounds, Vitamin E, and Fatty Acids Compositional Profiles in Black Seed-Coated Soybeans (*Glycine Max* (L.) Merrill) depending on Pickling Period in Brewed Vinegar**

Ill-Min Chung, Jin-Young Oh, and Seung-Hyun Kim*

Department of Crop Science, College of Sanghuh Life Science, Konkuk University, Seoul 05029, Republic of Korea

***the submitting (corresponding) author**: To whom correspondence should be addressed “Department of Crop Science, College of Sanghuh Life Science, Konkuk University, 120 Neungdong-ro, Gwangjin-gu, Seoul 05029, Republic of Korea, Tel: +82-02-2049-6163; Fax:+82-02-455-1044; E-mail: [kshkim@konkuk.ac.kr](mailto:kshkim@konkuk.ac.kr)”

**Institution for all authors is same as Konkuk University.**

**Ill-Min Chung (IMC):** [imcim@konkuk.ac.kr](mailto:imcim@konkuk.ac.kr),

**Jin-Young Oh (JYO):** [jinyoung7327@naver.com](mailto:jinyoung7327@naver.com),

**Seung-Hyun Kim (SHK):** kshkim@konkuk.ac.kr

**Table S1.** Concentration range, linearity, limit of detection, and limit of quantification of 23 phenolic standards examined in this study

| Group | Standard | Concentration range^a^ (μg·mL^-1^) | Linearity (r^2^) | Slope | SD of y-intercept | LOD ^b^ (μg·mL^-1^) | LOQ ^b^ (μg·mL^-1^) |  |
| --- | --- | --- | --- | --- | --- | --- | --- | --- |
| Phenolic acid  (12) | Gallic acid | 0.5 – 10 | 0.99 | 3.99 | 0.58 | 0.44 | 1.46 |  |
|  | Protocatechuic acid | 1 – 10 | 0.99 | 2.62 | 0.18 | 0.21 | 0.70 |  |
|  | Gentisic acid | 0.5 – 10 | 0.99 | 0.24 | 0.05 | 0.58 | 1.94 |  |
|  | 4-hydroxy benzoic acid | 0.5 – 10 | 0.99 | 2.85 | 0.25 | 0.27 | 0.89 |  |
|  | Syringic acid | 0.5 – 10 | 1 | 5.67 | 0.14 | 0.07 | 0.24 |  |
|  | Chlorogenic acid | 0.5 – 10 | 1 | 2.98 | 0.13 | 0.13 | 0.45 |  |
|  | *p*-coumaric acid | 0.5 – 10 | 0.99 | 9.98 | 0.69 | 0.21 | 0.69 |  |
|  | Ferulic acid | 0.5 – 10 | 1 | 7.13 | 0.16 | 0.07 | 0.22 |  |
|  | *m*-coumaric acid | 0.1 – 2.5 | 0.99 | 12.18 | 0.20 | 0.05 | 0.17 |  |
|  | *o*-coumaric acid | 0.5 – 10 | 1 | 10.69 | 0.21 | 0.06 | 0.19 |  |
|  | *t*-cinnamic acid | 1 – 10 | 0.99 | 16.52 | 0.87 | 0.16 | 0.52 |  |
|  | Vanillin† | 1 – 25 | 0.99 | 7.27 | 0.61 | 0.25 | 0.84 |  |
|  | | | | | | | | |
| Flavonoid  (11) | Naringin | 1 – 10 | 0.99 | 3.09 | 0.15 | 0.14 | 0.49 |  |
|  | Catechin | 1 – 25 | 0.99 | 2.05 | 0.28 | 0.41 | 1.37 |  |
|  | Naringenin | 1 – 10 | 0.99 | 5.73 | 0.34 | 0.18 | 0.59 |  |
|  | Hesperetin | 0.5 – 10 | 0.99 | 5.74 | 0.62 | 0.33 | 1.08 |  |
|  | Rutin | 0.5 – 10 | 0.99 | 1.74 | 0.39 | 0.67 | 2.23 |  |
|  | Myricetin | 0.5 – 10 | 1 | 0.97 | 0.00 | 0.01 | 0.02 |  |
|  | Quercetin | 1 – 10 | 0.99 | 0.80 | 0.48 | 1.81 | 6.03 |  |
|  | Formonometin | 0.5 – 10 | 0.99 | 1.43 | 0.37 | 0.78 | 2.61 |  |
|  | Kaempferol | 0.5 – 10 | 1 | 1.58 | 0.03 | 0.05 | 0.18 |  |
|  | Biochanin A | 0.5 – 10 | 0.99 | 4.49 | 0.39 | 0.26 | 0.87 |  |
|  | Resveratrol‡ | 0.5 – 10 | 0.99 | 4.46 | 0.37 | 0.25 | 0.83 |  |

^†^Vanillin is phenolic aldehyde but separated by phenolic acid. ^‡^Resveratrol is stilbene but separated by flavonoid. ^a^Calibration curve was composed of 3 to 4 different concentrations for each standard solution. ^b^ LOD = 3 × SD/S, LOQ = 10 × SD/S, where SD is the standard deviation of the y-intercept of a calibration curve, and S is the slope of each calibration curve.

**Table S2.** Concentration range, linearity, limit of detection, and limit of quantification of vitamin E molecules examined in this study

| Group | type | Concentration range^a^ (μg·mL^-1^) | Linearity (r^2^) | Slope | SD of y-intercept | LOD^b^ (μg·mL^-1^) | LOQ^b^ (μg·mL^-1^) |
| --- | --- | --- | --- | --- | --- | --- | --- |
| tocopherol | α | 1–25 | 0.99 | 0.86 | 0.22 | 0.77 | 2.59 |
|  | β | 1–25 | 0.99 | 0.92 | 0.19 | 0.62 | 2.07 |
|  | γ | 1–100 | 0.99 | 0.82 | 0.12 | 0.50 | 1.70 |
|  | δ | 1–200 | 1 | 0.74 | 0.10 | 0.38 | 1.26 |
| tocotrienol | α | 1–10 | 0.99 | 1.04 | 0.08 | 0.24 | 0.80 |
|  | β | 1–10 | 0.99 | 0.13 | 0.12 | 1.12 | 3.75 |
|  | γ | 1–10 | 0.99 | 0.69 | 0.04 | 0.11 | 0.35 |
|  | δ | 1–10 | 0.99 | 1.01 | 0.11 | 0.48 | 1.62 |

^a^Calibration curve was composed of 3 to 4 different concentrations for each standard solution. ^b^ LOD = 3 × SD/S, LOQ = 10 × SD/S, where SD is the standard deviation of the y-intercept of a calibration curve, and S is the slope of each calibration curve.


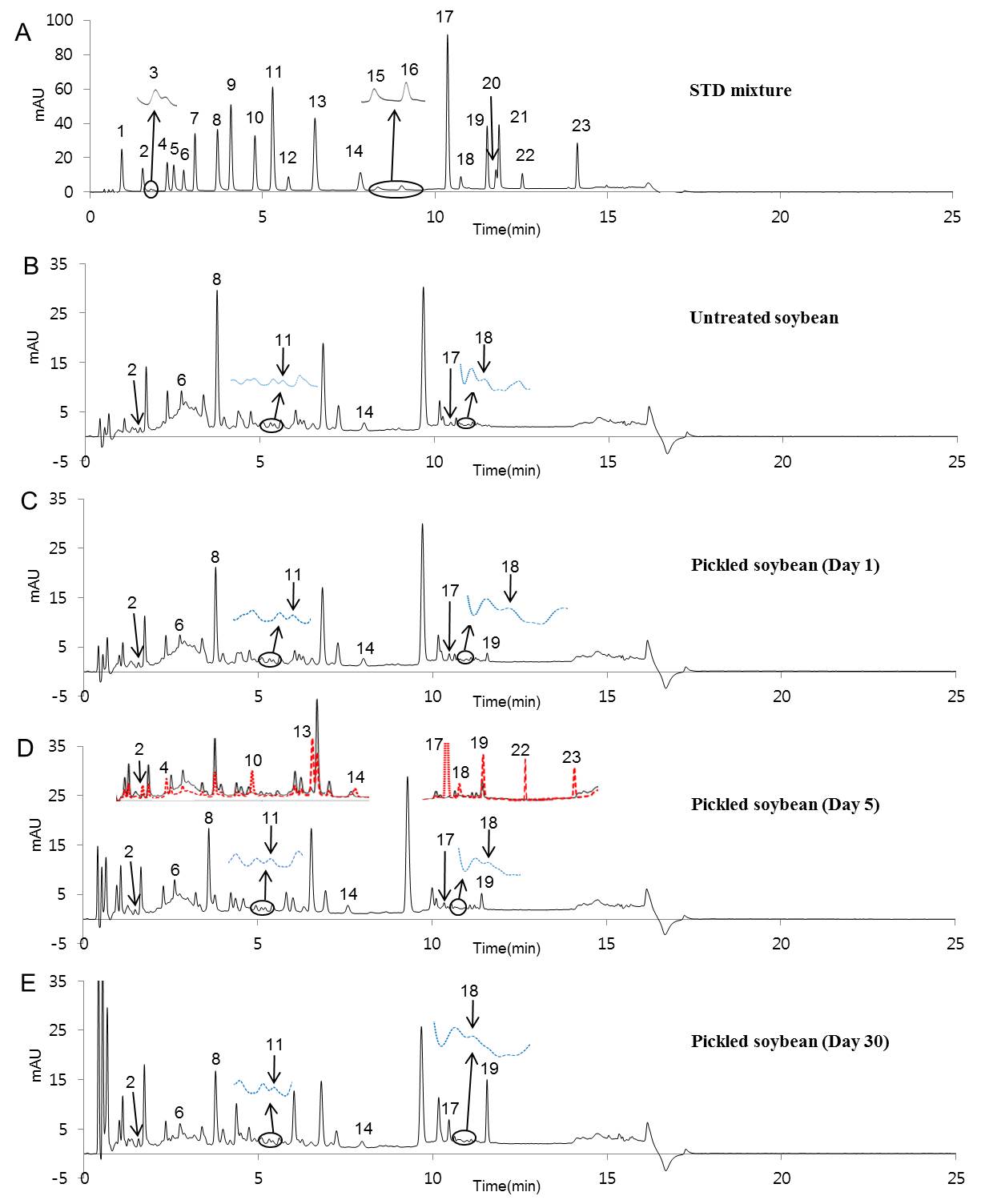


**Fig. S1.** **Representative UPLC chromatograms of phenolic compounds in the pickled soybeans (*p* < 0.05, *n* = 3).** A: 23 phenolic standards mixture; B: untreated soybean; C: pickled soybean at 1-day pickling process; D: pickled soybean at 5-day pickling process; E: pickled soybean at 30-day pickling process. The upper blue dot chromatograms are enlarged, and red dot chromatograms are the samples fortified (spiked) with certain phenolic standards. 1: gallic acid; 2: protocatechuic acid; 3: gentisic acid; 4: 4-hydroxy benzoic acid; 5: chlorogenic acid; 6: catechin; 7: syringic acid; 8: vanillin; 9: *p*-coumaric acid; 10: ferulic acid; 11: *m*-coumaric acid; 12: rutin; 13: *o*-coumaric acid; 14: naringin; 15: myricetin; 16: resveratrol; 17: *t*-cinnamic acid; 18: quercetin; 19: naringenin; 20: kaempferol; 21: hesperetin; 22: formononetin; 23: biochanin A


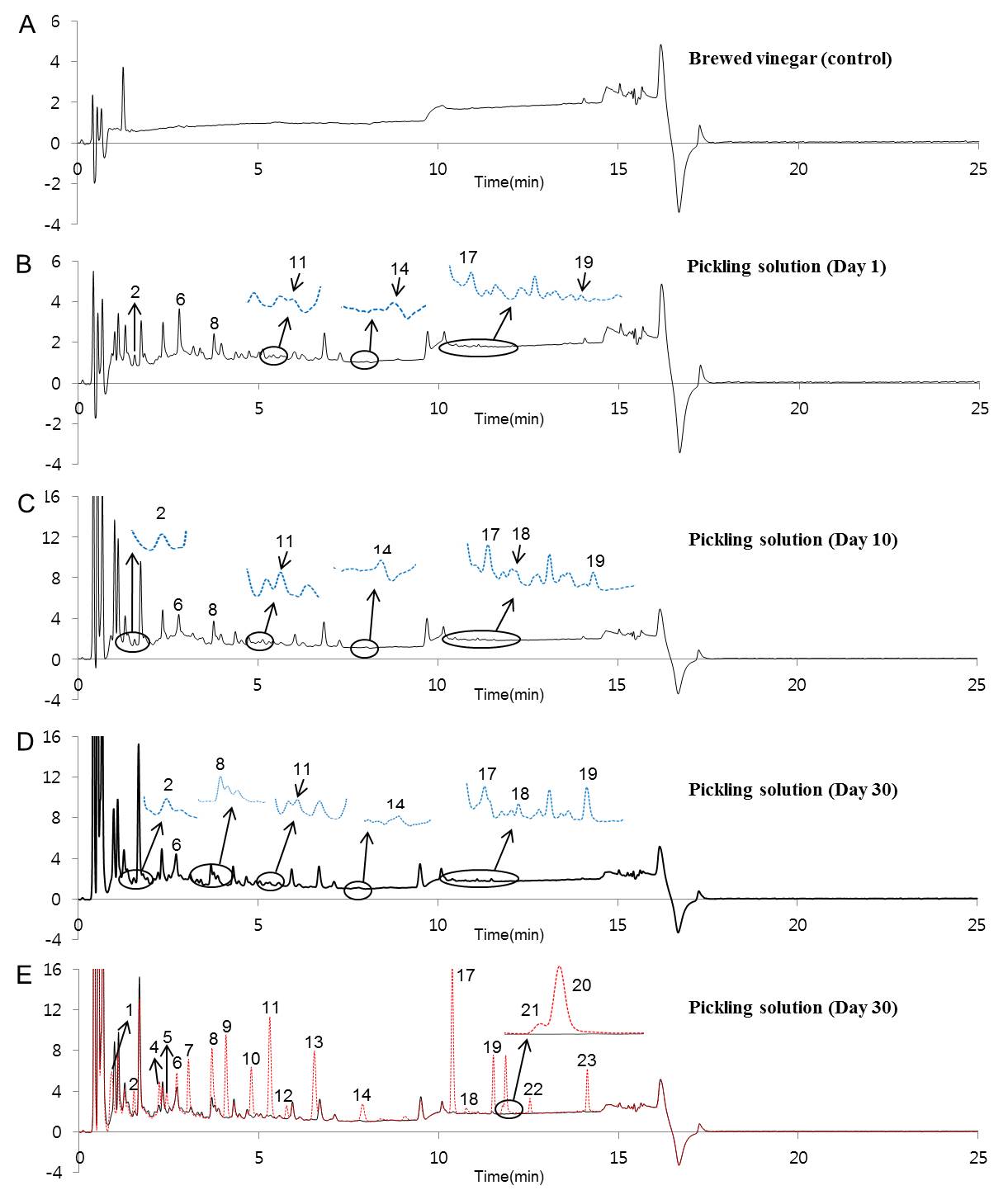


**Fig. S2.** **Representative UPLC chromatograms of phenolic compounds in pickling solution (*p* < 0.05, *n* = 3).** A: brewed vinegar used for the pickled soybean production in this study; B: pickling solution at 1-day pickling process; C: pickling solution at 10-day pickling process; D: pickling solution at 30-day pickling process; E: pickling solution fortified with phenolic STDs at 30-day pickling process. The upper blue dot chromatograms are enlarged, and red dot chromatograms are the samples fortified (spiked) with certain phenolic standards. 1: gallic acid; 2: protocatechuic acid; 3: gentisic acid; 4: 4-hydroxy benzoic acid; 5: chlorogenic acid; 6: catechin; 7: syringic acid; 8: vanillin; 9: *p*-coumaric acid; 10: ferulic acid; 11: *m*-coumaric acid; 12: rutin; 13: *o*-coumaric acid; 14: naringin; 15: myricetin; 16: resveratrol; 17: *t*-cinnamic acid; 18: quercetin; 19: naringenin; 20: kaempferol; 21: hesperetin; 22: formononetin; 23: biochanin A


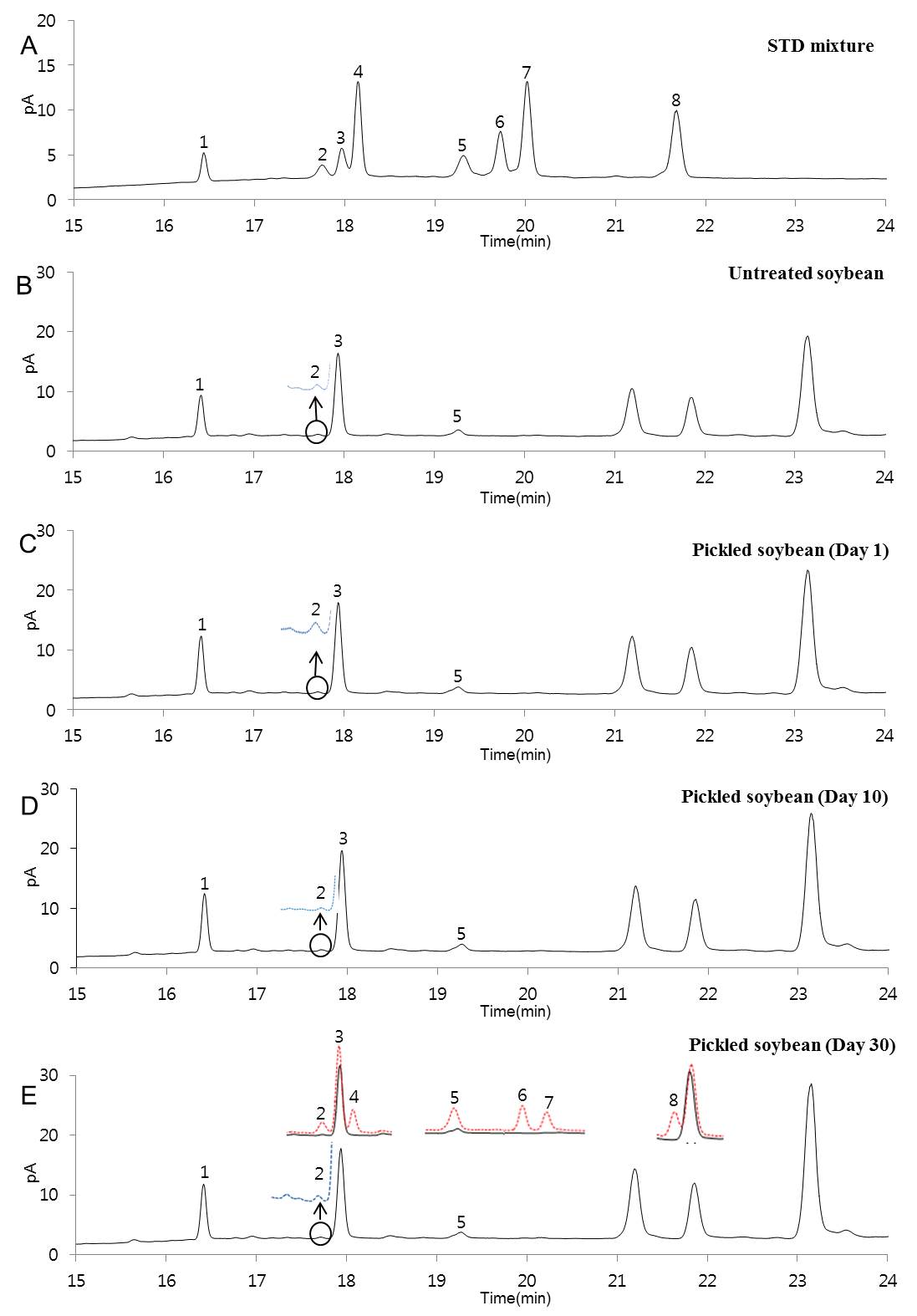


**Fig. S3. Representative GC-FID chromatograms of vitamin E in the pickled soybean.** A: 8 vitamin E standards; B: untreated soybean; C: pickled soybean at 1-day pickling process; D: pickled soybean at 10-day pickling process; E: pickled soybean at 30-day pickling process. The upper blue dot chromatograms are enlarged, and red dot chromatograms are the samples fortified (spiked) with certain phenolic standards. 1: δ-tocopherol; 2: β-tocopherol; 3: γ-tocopherol; 4: δ-tocotrienol; 5: α-tocopherol; 6: β-tocotrienol; 7: γ-tocotrienol; 8: α-tocotrienol


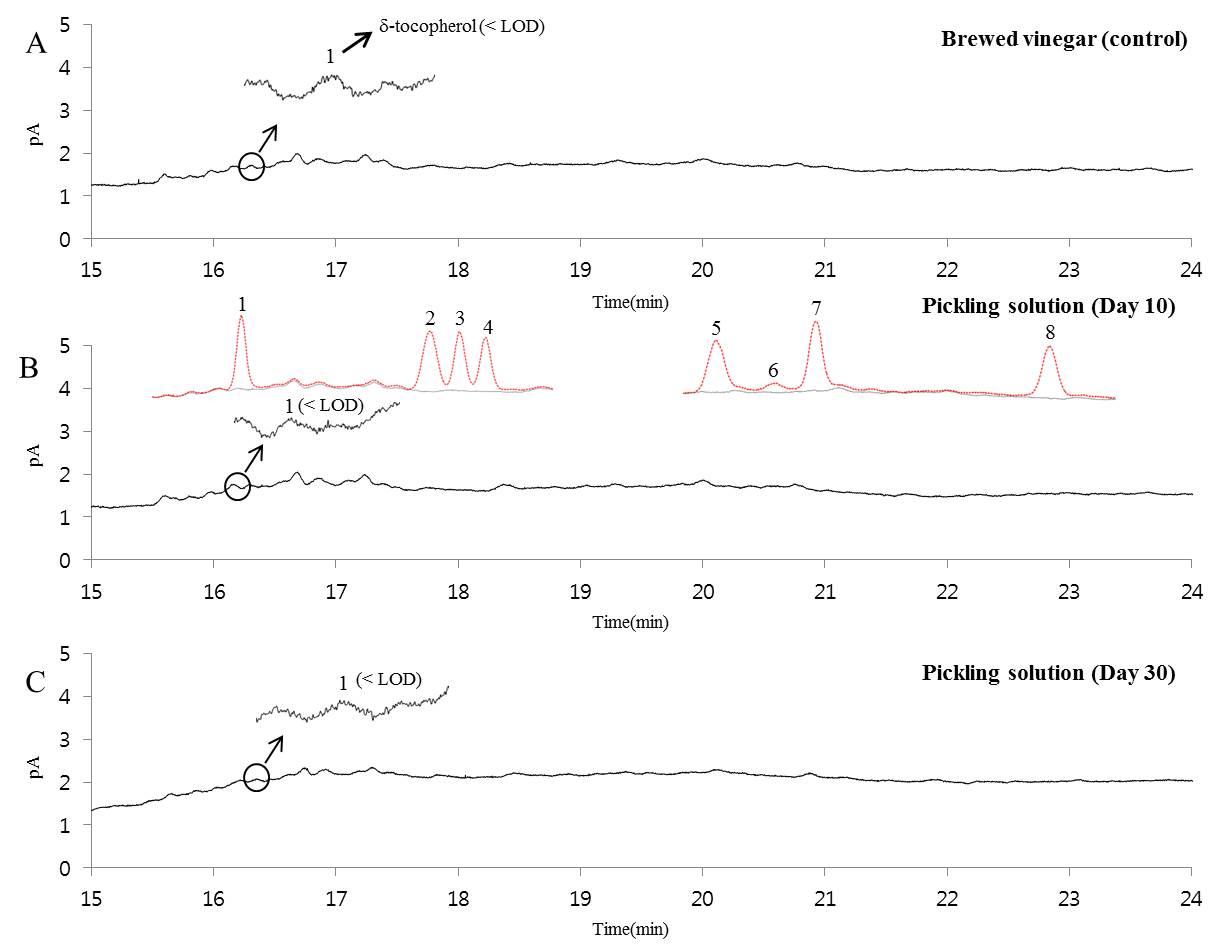


**Fig. S4. Representative GC-FID chromatograms of vitamin E in the pickling solution.** A: brewed vinegar; B: pickling solution at 10-day pickling process; C: pickling solution at 30-day pickling process. The upper blue dot chromatograms are enlarged, and red dot chromatograms are the samples fortified (spiked) with certain phenolic standards. 1: δ-tocopherol; 2: β-tocopherol; 3: γ-tocopherol; 4: δ-tocotrienol; 5: α-tocopherol; 6: β-tocotrienol; 7: γ-tocotrienol; 8: α-tocotrienol

**
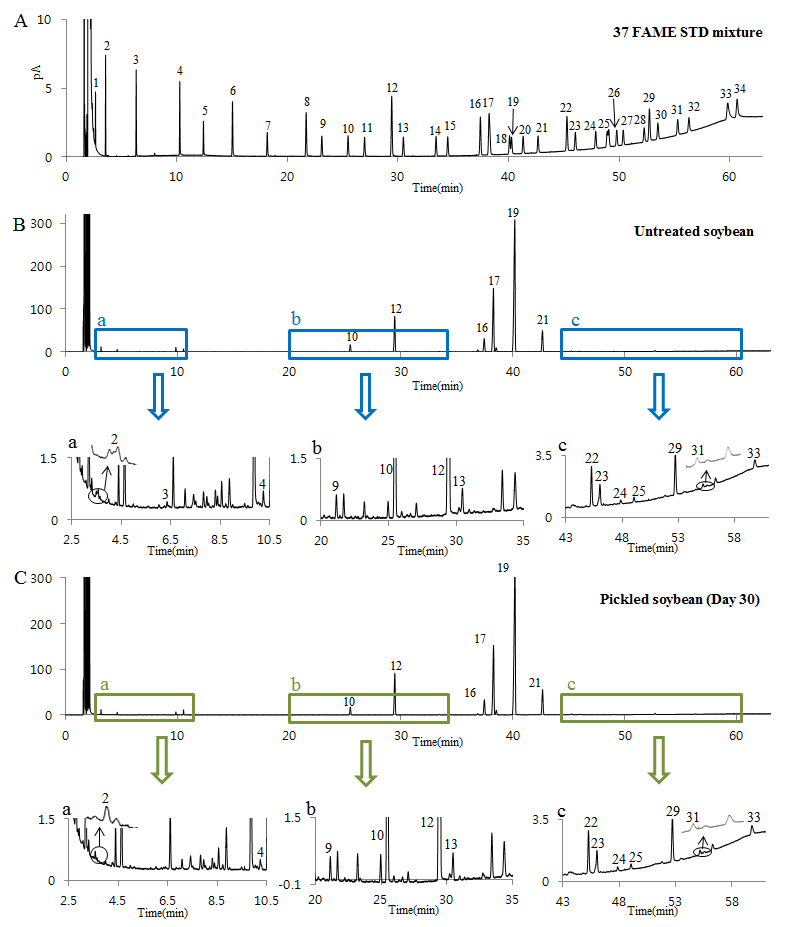
**

**Fig. S5. Representative GC-FID chromatograms of fatty acids in the pickled soybean.** A: 37 FAME STD mixture; B: untreated soybean; C: pickled soybean at 30-day pickling process; 1: C4:0; 2: C6:0; 3: C8:0; 4: C10:0; 5: C11:0; 6: C12:0; 7: C13:0; 8: C14:0; 9: C14:1; 10: C15:0; 11: C15:1; 12: C16:0; 13: C16:1; 14: C17:0; 15: C17:1; 16: C18:0; 17: C18:1 n-9 t and c; 18: C18:2 n-6 trans; 19: C18:2 n-6 cis; 20: C18:3 n-6; 21: C18:3 n-3; 22: C20:0; 23: C20:1 n-9; 24: C20:2; 25: C20:3 n-6 and C21:0; 26: C20:3 n-3; 27: C20:4 n-6; 28: C20:5 n-3; 29: C22:0: 30: C22:1 n-9; 31: C22:2; 32: C23:0; 33: C24:0; 34: C22:6 n-3 and C24:1 n-9
